# Mass Psychogenic Illness in Haraza Elementary School, Erop District, Tigray, Northern Ethiopia: Investigation to the Nature of an Episode

**DOI:** 10.1155/2020/2693830

**Published:** 2020-07-23

**Authors:** Kiros Fenta Ajemu, Tewolde Wubayehu Weldearegay, Nega Mamo Bezabih, Yrgalem Meles, Goytom Mehari, Abraham Aregay Desta, Asfawosen Aregay Berhe, Micheale Jorjo, Ataklti Gebretsadik Weldegebriel, Tesfay Subagadis Gebru, Abenezer Tesfadingle

**Affiliations:** ^1^Tigray Health Research Institute, Mekelle, Tigray, Ethiopia; ^2^Tigray Regional Health Bureau, Mekelle, Tigray, Ethiopia; ^3^Duhan Primary Hospital, Erop District, Eastern Tigray, Ethiopia; ^4^Erop District Health Office, Eastern Tigray, Ethiopia

## Abstract

**Background:**

Mass psychogenic illness has been documented for more than 600 years in a variety of cultural, ethnic, and religious settings. We aimed to assess the nature and characteristics of mass psychogenic illness and to evaluate community awareness and perception about the treatment they practiced in Haraza Elementary School, Erop district, Tigray, Northern Ethiopia.

**Methods:**

A school-based cross-sectional study was conducted in Haraza Elementary School from January to February, 2020. Students who were victims of an episode were subjects of the study. A total of twelve students were investigated using a semistructured questionnaire for a quantitative study. Seven key informant interviews were conducted using a guiding questionnaire. Quantitative data was analyzed using XL sheet while qualitative data were analyzed manually.

**Results:**

The mean age of study participants was 14 years (SD ± 1.3). The majority (87%) were teenage female students. The incident was an unspecified disease with psychiatric disorder, migraine, and syncope with no plausible organic causes. An important feature of migraine and syncope was their comorbidity with mass psychogenic illness. The community perceived that evil devil force and blaming the being as an evil eye were common causes of the occurrence of an episode.

**Conclusion:**

Lack of empirical knowledge and awareness about its management and prevention among community members and health professionals resulted exaggerated rumor that would perceive as newly emerging disease that affected school activities. Integrating MPI in PHEM package at health facility level, advocacy workshops for media, and other relevant stakeholders will minimize its impact for the future.

## 1. Introduction

Mass psychogenic illness (MPI) or mass hysteria is not a rare phenomenon around all corners of the world. It has been defined as a group of physical signs and symptoms that suggest the presence of organic illness, but without any clinical and laboratory evidence of disease [1, 2]. Since the year 1374, it has been documented for more than 600 years in a variety of culture [3, 4], ethnic [5], and religious [6, 7] settings.

There is no conclusive evidence about the causes of the illness, but psychological factors, environmental factors, different stresses, conflicts, lower level of education, lower socioeconomic status, minority race, and history of abuse or trauma were commonly postulated causes [8, 9]. It is common in rural areas among uneducated people, lower socioeconomic classes, and ethnic minority groups. It is usually seen in girls and teenager groups. The episode prolonged from weeks to months [10, 11].

Evidences from various studies revealed that it commonly affects people who live in groups: schools (50%), town and villages (10%), factories (29%), and family groups (4%). Sometimes, it is notified from nunneries, boarding houses, prisons, and religious institutions [5, 12, 13].

The majority of the outbreaks were recognized due to environmental “trigger” such as bad smell, abnormal sound, a suspicious looking substance, or something else that makes members of the cohesive group exposed to a danger [14]. It is typically begun when an individual with an index case becomes ill hysterically during a period of stress followed by multiple people experiencing of the same symptoms [5, 13]. Among the total reported hospitalized ill students in Bangladesh, 88% complain about consumption of cake with abnormal smell or taste. Among these, 20% of them felt ill by only seeing other ill students in the school [15]. Satanism and evil devil force, punishment by God, due to the presence of toxic chemicals, polluted environment, cold air, and using family planning injection or pills had been discovered around the schools and community members in Ethiopia [15, 16].

In Africa, evil spirit, witchcraft, Satanism, or failures to perform cultural and religious rituals have been mentioned as the causes of the illnesses [5]. Similar finding was reported from Kombolcha of Northern Ethiopia and Derashe of Southern Ethiopia [9, 17]. On the other hand, toxic chemicals and environmental pollution have been notified in western setting: India due to toxic fumes [14]; these attributions in Africa lead many victims to commonly seek treatment from different religious and traditional healing sites [14, 17]. This is also practiced in the Ethiopia context in which more than half (63.9%) of the study participants in Derashe district of Southern Ethiopia reported that they had been visiting traditional healing services; 8.2% had sought treatment from religious services, modern health service (75.3%), and all the three treatment sites (5.1%), namely, traditional healers, religious healers, and health institutions [17].

It is recognized as a rapid spread of illness, signs, and symptoms mainly affecting members of a cohesive group that has been originating from a nervous system disturbance involving excitation, loss, or alteration of function, whereby physical complaints that are exhibited unconsciously have no corresponding organic etiology [2].

Common symptoms of mass psychogenic illness reported were nausea, dizziness, fainting, headache, abdominal pain, hyperventilation, cough, fatigue, drowsiness, weakness, watery eyes, chest pain, vomiting, communication difficulties, laughing, and fainting which were common in African settings [5, 8–10, 15].

Mass psychogenic outbreaks were reported in different African settings, including Ethiopia [5, 13, 15]. Similar outbreaks of MPI were also reported in different Ethiopian settings: Bati, Kombolcha, Derashe, and Gondar [8, 9, 16, 17]. Despite the difference in culture, religion, and age range, the clinical presentation of the cases during an episode was similarly reported. All reported incidents brought a common intense of a public and community terror to the extent of collapsing school and occupational activities. Though there is an increase in awareness among health professionals, it is still under considered, under-reported, and still causing significant health and social problems in the country.

However, epidemics of hysteria are under-reported or not studied well across the country. Besides, none was conducted in the study region in Tigray region, Northern Ethiopia. The incident happened for the first time in Eastern parts of Tigray, Northern Ethiopia in Erop district. Its clinical characteristics, community perception about the root cause, treatment, and its prevention mechanism were not known. Therefore, we aimed to assess the nature and characteristics of mass psychogenic illness in Haraza Elementary School, Erop district, Tigray, Northern Ethiopia.

## 2. Methods

### 2.1. Study Design and Settings

A school-based cross-sectional study was conducted from January to February 2020 in Erop district, Tigray, Northern Ethiopia. The district administrative setup was composed of 35,000 people [18]. A mixed-method approach was used involving both quantitative and qualitative data collection methods.

### 2.2. Sampling and Sample Size Determination

A total of twelve complaints of unknown disease were enrolled in the study. For quantitative data, a standardized [17] semistructured questionnaire was used to review the nature and characteristics of the disease from hospitalized patients. A total of 18 complaints were reported from the community, but only 12 of them were found during the data collection period and attending health service in a nearby health facility. However, the remaining six were somewhere else in holy waters and not easily contacted during data collection. A convenience sampling method was used to select participants for qualitative data in which seven key informant interviews (KIIs) were subjected for key informant interview. The inclusion criteria for enrolling KIIs were their availability in the school during an event and their past experience of similar events. Participants were enrolled from community members, health professionals, and school members.

#### 2.2.1. Inclusion Criteria

Participants enrolled for quantitative data were victims of an episode who received health ervice during the onset in the nearby health facility and available in the community during the data collection period, whereas respondents subjected for KIIs were those that observed the event and had an exposure for a similar event in the past.

#### 2.2.2. Exclusion Criteria

The exclusion criteria are those victims not available in the community and who were in holy water during data collection period.

### 2.3. Data Collection Procedure

A record review of sociodemographic characteristics, symptoms of illness, and onset and duration of illness was conducted from a line list for quantitative data. In some cases, card review was done to summarize the patient history and other investigations done during hospital admission in order to rule out recent underlying medical illnesses and organic causes. Key informant interviews were conducted using a guiding questionnaire adopted from a similar study conducted in Dershe district of Southern Ethiopia [17]. Home to home interviews were conducted with families of the index case and community members until information saturation was obtained. Participants were interviewed alone to secure the confidentiality and to avoid information bias. Quantitative data were collected by a physician working in Duhan District Primary Hospital while a principal investigator conducted key informant interviews.

## 3. Data Analysis and Management

Quantitative data were entered and analyzed using excel 2007 spread sheet. Descriptive data were used to illustrate the prevalence of an incident among hospitalized patients. Verbatim transcription of KIIs was done, and transcriptions were translated from “Eropigna” to “Tigrigna” and back to English. Some of them were translated directly from “Tigrigna” to English. We used thematic analysis [19]. Qualitative data analysis involved reading scripts several times, identifying themes and subthemes, and grouping data for interpretation [19, 20]. Transcriptions were read multiple times to validate transcription and familiarize with predetermined study objectives and themes. Preliminary themes were prepared by categorizing data into groupings. Key quotes were selected and classified concurrently to clarify interviewees' perception about the incident. Preliminary themes were refined, and finally, groups of related data were clustered to similar themes and categories. Quotes that best described main themes were chosen and presented.

## 4. Results

### 4.1. Sociodemographic Characteristics

Among the total 12 study participants, majority (87%) were teenage female students. The age of victims of the episode ranged from 13 to 16 years with a mean of 14 years (SD ± 1.3). All participants were from Haraza kebelle where an index case has been living.

### 4.2. Onset and Duration of an Episode

**Figure 1:**
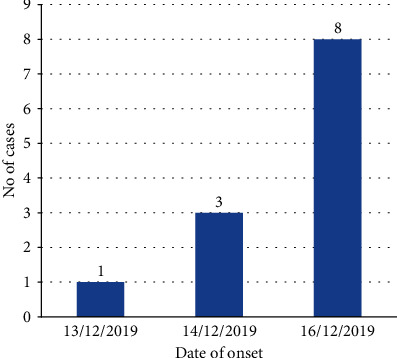
Onset of mass psychogenic illness in Haraza Elementary School, Tigray, Northern Ethiopia, 2020.

The incident was reported on the 13th of December 2019, when a 13-year-old girl had been complaining about unknown disease. New cases were continued up to the 16th of December 2019 reaching a total of 12 cases (Figure 1). The duration of an illness ranged from 1 to 3 days, with a mean of 2 (SD ± 0.8) days.

### 4.3. Clinical and Laboratory Investigations

Major clinical symptoms and signs observed during hospital admission were inability to talk (75%), shouting (66.7%), dizziness (58.3%), headache (58.3%), and sleepiness (58.3%) (Table 1). However, the clinical findings showed normal vital signs (the average blood pressure ranged from 70 to 108/67 to 74 mmHg; pulse rate, from 73 to 98 beats per minute; body temperature, from 36 to 37.4°C, and respiratory rate, from 16 to 24 per minute). Besides, laboratory investigations were done for hospitalized cases. Findings revealed that Widal's test was nonreactive, hemoglobin range was 13-14 Mg/dl, blood film has no hemoparasite seen, and random blood sugar range was 97-304 Mg/dl. Having these realities, the clinician ruled out no plausible organic caused illness, and he categorized the incident as an unspecified disease with psychiatric disorder, migraine headache, and syncope.

Seven key informant interviews were conducted with a parent of index case, a community member, a school principal, a teacher who was teaching while an incident was occurring, a health extension worker, a surveillance officer, and a physician managing an incident in hospital (Table 2). The mean age of study participants was 43(SD ± 12.6). The information gathered from the interview was categorized in the following five themes: onset of an episode, community perception about the incident, treatment sought by the community, health professional awareness, and psychosocial impacts of the illness (Table 2).

### 4.4. Theme 1: Onset of an Episode

During an interview, the incident was reported for the first time. The onset of illness was traced following the index case of a 13-year-old student complaining about unknown disease while she was learning at school as expressed by her teacher: “I had been teaching English for grade 6^th^ after a while index cases was disappointed and asking me to be out from the class and I am letting her to be out with her three class mates. After a while they also felt uncomfortable and started showing similar symptom with her” (KII ≠2).

As the news spread among other students, the number of cases started increasing and became a total of twelve victims within three consecutive days. Then, the school was decided to be closed when an agreement was reached by school members, community, and political leaders as there were high community frustration and psychosocial disturbance: “because of the incident, students fear of being affected by the illness considering it as communicable disease lead a schools to be closed for three days and a number of students were recorded as dropout from school” (KII ≠1).

### 4.5. Theme 2: Community Perception about the Episode

During an interview, no specific cause of the incident was forwarded. However, a common manifestation was characterized by irrational behavior or beliefs. The majority of the participants of the interview illustrated that the triggering risk factor was an evil devil force other than an environmental factor (see Table 2). Two informants expressed their own concern about community perception about its root cause: “this was a new incident and we never experienced with such an event but this might be due to Satanism and an Evil devil force that came in line with the new political reform in the country” (KII ≠2) and “families of the patient informed me not to provide treatment with injection since our students were ill due to the presence of evil-eyed teacher in the school” (KII ≠2).

### 4.6. Theme 3: Treatment Sought by the Community

During an interview, a common treatment practice sought by the community during the incident was a frequent follow-up of traditional and religious healers rather than attending a modern health service: “majority of the complaints were practicing and visiting Holly-Water, sorcerer, and praying for ‘God' to treat the disease” (KII ≠3).

### 4.7. Theme 4: Health Professional Awareness

During the discussion with informants, community members reported that they are not confident enough by health professionals since no clear and an adequate explanation was given during their visit: “I was attending health service in different health institutions but not adequate explanation was given for me about the nature of disease that why I prefer to attend traditional religious service “(KII ≠2).

### 4.8. Theme 5: Psychosocial Impacts of the Illness

The incident was perceived as a contagious communicable disease; as a result, the community was not reluctant to send their children to school. Similarly, students were also not delighted to attend the school: “many students were in fear of the outbreak perceiving it as a communicable disease and they were frustrated to attend teaching learning process” (KII ≠2).

## 5. Discussion

This was the first regional mass psychogenic of regional outbreak investigation. The incident was an unspecified disease with psychiatric disorder, migraine, and syncope with no plausible organic causes. An important feature of migraine and syncope was their comorbidity with mass psychogenic illness.

Based on this finding, students were suffering from mass psychogenic illness (MPI) for the following reasons: the incident was categorized as with no plausible organic cause of illness, since findings from a clinical investigation of an episode were unspecified disease with psychiatric disorder, migraine headache, and syncope. An important feature of migraine and syncope was their comorbidity with mass psychogenic illness and other neurological diseases [21–24]. A similar incident was reported from South Africa and Malawi [25, 26] but unlike the current incident due to infectious disease which was reported from the village of India, West Bengal, and North Carolina [6, 27, 28]. Besides, consumption of cake in Bangladesh was noticed by 88% of ill students [21], sociocultural beliefs like devil-evil forces and religious ritual evil force in Taiwan [22].

Similar to this study, various studies published between 1973 and 1993 showed that schools are a very common place for mass psychogenic illness, accounting more than 50% of the events [8, 29]. This was similarly evidenced from [30, 31], South Africa [32], and Ethiopia [8, 9]. Similarly, the majority (82%) of the affected cases were females. A similar type of outbreaks with a frequent attack of teenage female groups was reported from similar study settings [8, 9, 33]. Such outbreak led a very rapid spread of symptoms which frequently includes chilling, fear, crying and shouting, anxiety, abdominal pain, communication difficulties, laughing, generalized weakness, muscle cramp, and fainting which is consistent with other similar studies [5, 8, 9, 15, 17].

The majority of the affected women were from Haraza kebelle where the index case had lived. The duration of the outbreak stayed with an average of two days in which typically MPI affects the groups for limited time periods [8]. As to the causes of the illness, a majority of community members were confused even health professionals. However, findings from a qualitative study indicated community perception about the cause of the illness evil devil force [5, 9, 15, 17] and blaming the being as an evil eye [8], but the new political reform was a uniquely identified cause in this study. Unlike the current study, other outbreaks were attributed by environmental triggers [16, 25, 26, 34].

Prompt public awareness of an episode of mass psychogenic illness has been an important step found helpful to control its spread and reemergence [35]. Among the crucial steps in managing MPI are as follows: consider involving a behavioral scientist, psychologist, or psychiatrist experienced in this area and try to minimize the persistence of rumors and media reports, which can trigger relapses of new cases, by giving out clear health messages [2, 35].

We also recommend that including MPI in PHEM package at health facility level enables PHEM officers to be aware with such newly emerging outbreak and easily managing it. Advocacy workshops of this type of outbreaks for the community, media professionals, and other relevant stakeholders are also essential to avoid unrealistic and exaggerated rumor reports that increase community frustration.

### 5.1. Strength and Limitation of the Study

The strength of the study was the involvement of a mixed-method study supported by laboratory investigations that increase the fidelity and validity of the study. Despite this strength, the study uses a small sample size, which might be considered a limitation.

## 6. Conclusion

The lack of empirical knowledge and awareness about its management and prevention among community members and health professionals resulted in exaggerated rumor that would perceive as newly emerging disease that leads to community frustration and social disturbance which facilitate school dropout. Integrating MPI in PHEM package at health facility level, advocacy workshops for media and other relevant stakeholders, and introducing appropriate and timely strategies should be designed that will minimize its impact for the future.

## Figures and Tables

**Table 1:** Common reported symptoms of mass psychogenic illness in Haraza Elementary school, Tigray, Northern Ethiopia, 2019 (*n* = 12).

Categories	Number	Percent
Headache	7	58.3
Dizziness	7	58.3
Unable to talk	9	75
Loss of appetite	6	50
Low-grade fever	3	25
Abnormal body movement	3	25
Cough	3	25
Sleepiness	7	58.3
Shouting	8	66.7
Laughing	3	25
Unconsciousness	6	50
Breathlessness	6	50
Generalized weakness & fatigue	6	50

Findings from qualitative data.

**Table 2:** Summery of themes and subthemes for the occurrence of mass psychogenic illness in Haraza Elementary School, Tigray, Northern Ethiopia, 2019.

Category (CAs)	Themes (TMs)	Orders/subthemes (Os/ST)
School community members	Theme 1: onset of an episode	Onset of illness occurred for the first time
		Incident occurred while students were learning English
		Index case does not have an economic problem
		Index case was a middle-level student
		No one told us the type of incident even health workers
Community members	Theme 2: community perception about the incident	Such type of incident does not occur before in the locality
Health professionals		
		No one told us the type of incident including health professionals
		Such type of incident does not occur before in the locality
		No one told us the type of incident including health professionals
		Unknown communicable disease
		Blaming of an unidentified teacher being an evil eye
Community members	Theme 3: treatment sought by the community	Frequent follow-up of holy water
		Visiting a sorcerer
		Praying for “God”
		Not confident to attend the health service
		The disease not known by health professionals
Health professionals	Theme 4: health professional awareness	We do not know the disease and did not experience before
		The disease was unspecified
		It was difficult for us to manage the incident
Health professionals	Theme 5: psychosocial impacts of the illness	Discriminating complaints thinking being transmitted disease
Community members		School dropout
		Community frustration not sending students at school

## Data Availability

The datasets used and/or analyzed for the study were available from the corresponding author on reasonable request.

## Abbreviations

MPI: Mass psychogenic illness
PHEM: Public Health Emergency Management
KII: Key informant interview.
